# The pathogenesis and therapeutic implications of metabolic reprogramming in renal cell carcinoma

**DOI:** 10.1038/s41420-025-02479-9

**Published:** 2025-04-19

**Authors:** Yifan Zhang, Shengli Zhang, Hongbin Sun, Luwei Xu

**Affiliations:** Department of Urology, Nanjing First Hospital, Nanjing Medical University, Nanjing, Jiangsu People’s Republic of China

**Keywords:** Cancer metabolism, Renal cell carcinoma

## Abstract

Renal cell carcinoma (RCC), a therapeutically recalcitrant genitourinary malignancy, exemplifies the profound interplay between oncogenic signaling and metabolic adaptation. Emerging evidence positions metabolic reprogramming as a central axis of RCC pathogenesis, characterized by dynamic shifts in nutrient utilization that transcend canonical Warburg physiology to encompass lipid anabolism, glutamine auxotrophy, and microenvironment-driven metabolic plasticity. This orchestrated rewiring of cellular energetics sustains tumor proliferation under hypoxia while fostering immunosuppression through metabolite-mediated T cell exhaustion and myeloid-derived suppressor cell activation. Crucially, RCC exhibits metabolic heterogeneity across histological subtypes and intratumoral regions—a feature increasingly recognized as a determinant of therapeutic resistance. Our review systematically deciphers the molecular architecture of RCC metabolism, elucidating how VHL/HIF axis mutations, mTOR pathway dysregulation, and epigenetic modifiers converge to reshape glucose flux, lipid droplet biogenesis, and amino acid catabolism. We present novel insights into spatial metabolic zonation within RCC tumors, where pseudohypoxic niches engage in lactate shuttling and cholesterol efflux to adjacent vasculature, creating pro-angiogenic and immunosuppressive microdomains. Therapeutically, we evaluate first-in-class inhibitors targeting rate-limiting enzymes in de novo lipogenesis and glutamine metabolism, while proposing biomarker-driven strategies to overcome compensatory pathway activation. We highlight the synergy between glutaminase inhibitors and PD-1 blockade in reinvigorating CD8^+^ T cell function, and the role of lipid-loaded cancer-associated fibroblasts in shielding tumors from ferroptosis. Finally, we outline a translational roadmap integrating multi-omics profiling, functional metabolomics, and spatial biology to match metabolic vulnerabilities with precision therapies.

## Facts

- Metabolic reprogramming enables tumor cells to survive within the tumor microenvironment, circumvent cellular stress responses, and resist therapeutic interventions.
- Metabolic reprogramming plays a pivotal role in the development of renal cell carcinoma and its resistance to immune responses.
- Metabolic reprogramming holds promise as a therapeutic target for treating renal cell carcinoma.

## Open questions

- What are the distinct molecular mechanisms underlying various forms of metabolic reprogramming in renal cell carcinoma?
- Are the various types of metabolic reprogramming interconnected or synergistic in renal cell carcinoma?
- What are the unresolved challenges and complexities associated with leveraging metabolic reprogramming for the treatment of renal cell carcinoma?

## Introduction

Renal cell carcinoma (RCC), a malignancy arising from renal tubular epithelial cells, represents 2–3% of global cancer diagnoses and 85% of kidney neoplasms [1, 2]. Clear cell RCC (ccRCC), characterized by von Hippel-Lindau (VHL) tumor suppressor gene inactivation in >80% of cases, dominates the histological spectrum (75% of RCCs), followed by papillary (10–15%) and chromophobe (5%) subtypes [3, 4]. The insidious nature of early-stage RCC contributes to delayed diagnosis, with 25–30% of patients presenting metastatic disease at initial evaluation. While surgical resection (partial/radical nephrectomy) remains curative for localized tumors, 30–40% develop recurrence—a statistic underscoring the limitations of current surveillance strategies [4, 5]. Metastatic RCC (mRCC) portends dismal outcomes, with median overall survival (OS) historically ≤12 months [6, 7]. The advent of vascular endothelial growth factor receptor (VEGFR) tyrosine kinase inhibitors (TKIs) and immune checkpoint inhibitors (ICIs) has extended median OS to 24–30 months, yet durable responses remain elusive in 60–70% of patients due to clonal heterogeneity and adaptive resistance mechanisms [8–12].

At the nexus of RCC pathogenesis lies metabolic reprogramming—an evolutionary hallmark enabling cancer cells to rewire nutrient acquisition and utilization, thereby sustaining proliferation amidst microenvironmental stressors [13–15]. This metabolic plasticity transcends Otto Warburg’s century-old observation of aerobic glycolysis (the “Warburg effect”), encompassing VHL/hypoxia-inducible factor (HIF)-driven pseudohypoxia, mechanistic target of rapamycin (mTOR)-mediated anabolic activation, and epigenetic regulation of nutrient transporters [16–18]. ccRCC exemplifies this complexity: HIF-2α stabilization induces glycolysis via pyruvate dehydrogenase kinase (PDK)-mediated suppression of oxidative phosphorylation (OXPHOS), while simultaneously upregulating glutamine transporters to fuel reductive carboxylation—a metabolic bypass sustaining lipid biosynthesis under hypoxia [19, 20]. These adaptations generate an immunosuppressive tumor microenvironment (TME) through lactate accumulation (extracellular pH <6.5), kynurenine-mediated T cell exhaustion, and adenosine-driven myeloid suppression [21–24].

Emerging paradigms position metabolic crosstalk as a therapeutic amplifier. Preclinical models demonstrate that 3-bromopyruvate (glycolysis inhibitor) synergizes with anti-PD-1 therapy by reversing lactate-driven M2 macrophage polarization [25], while glutamine antagonists sensitize tumors to ferroptosis inducers by depleting glutathione precursors [26]. Clinically, the RECORD-3 trial revealed mTOR inhibitor everolimus preferentially benefits ccRCCs with TSC1/2 mutations—a testament to biomarker-driven metabolic targeting [27]. This review delineates the multifaceted roles and molecular mechanisms underpinning diverse metabolic reprogramming events in RCC, while critically evaluating their clinical translational potential. By systematically synthesizing emerging evidence, this work aims to provide novel perspectives for advancing fundamental research on RCC pathogenesis and informing future directions in clinical management strategies.

## Reprogramming mechanisms of metabolism in malignant tumors

Metabolic reprogramming has emerged as a central hallmark of malignancy, orchestrating tumor initiation and progression through dynamic adaptations that sustain proliferation amidst microenvironmental constraints [28–30]. This metabolic plasticity manifests through coordinated rewiring of glucose, amino acid, lipid, and nucleotide metabolism, creating biochemical profiles distinct from normal tissues. The Warburg effect exemplifies this phenomenon, where cancer cells preferentially engage glycolysis for ATP production and biosynthetic precursor generation despite oxygen availability, resulting in lactate accumulation that acidifies the TME and paralyzes anti-tumor immunity [31–33].

The mTOR pathway serves as a metabolic nexus, integrating nutrient sensing with anabolic processes through dual regulation of protein/lipid synthesis and catabolic suppression [34, 35]. mTORC1 activation stimulates SREBP-1-mediated fatty acid production while suppressing AMPK-driven lipid oxidation, creating a pro-synthetic state exploited by proliferating tumor cells [36, 37]. Counterbalancing this, TP53 maintains metabolic homeostasis by modulating OXPHOS efficiency and glutaminolysis—a safeguard frequently circumvented in malignancies through TP53 mutations [38, 39]. Myc amplifies these effects by globally upregulating glycolytic and glutamine catabolic genes, establishing feedforward loops that lock tumors into metabolic dependency.

Amino acid metabolism is profoundly remodeled, with glutamine emerging as a nitrogen/carbon shuttle fueling TCA cycle anaplerosis and redox homeostasis via α-ketoglutarate generation [40, 41]. BCAA catabolism similarly supports tumor anabolism, with branched-chain aminotransferase 1 (BCAT1) overexpression linking tissue-specific mutations to nitrogen flux in gliomas and leukemias [42–45]. Lipid reprogramming extends beyond mere energy storage-de novo fatty acid synthesis via ACLY generates substrates for post-translational modifications (e.g., palmitoylation) that regulate oncogenic signaling, while lipid droplets buffer oxidative stress and chemotherapeutic toxicity [46, 47].

Metabolic crosstalk within the TME creates therapeutic vulnerabilities. CAFs secrete lactate and ketone bodies that fuel OXPHOS in adjacent tumor cells, while TILs starved of glucose and glutamine exhibit impaired cytotoxic function [48]. Pharmacological disruption of this symbiosis-through LDHA inhibition to normalize pH or CAF-targeted silencing-synergizes with PD-1 blockade to restore immune surveillance [49, 50]. Emerging strategies exploit these interdependencies: glutaminase inhibitors potentiate ferroptosis in SLC7A11-high tumors, while mTORC1/2 dual antagonists overcome compensatory lipid scavenging in VHL-mutant ccRCC [51, 52].

## The impact of metabolic reprogramming on the occurrence and development of tumors

Metabolic reprogramming is one of the important mechanisms in the occurrence and development of tumors, which can regulate the biological behaviors such as growth and metastasis of malignant tumor cells, thereby affecting the development and therapeutic effects of tumors.

### Metabolic reprogramming impacts tumor growth and proliferation

Metabolic reprogramming stands as a defining feature of cancer pathogenesis, enabling malignant cells to circumvent microenvironmental constraints through dynamic adjustments in nutrient acquisition and utilization. Central to this adaptation is the Warburg effect, where tumor cells prioritize aerobic glycolysis over OXPHOS for ATP generation, despite oxygen availability—a strategy that not only accelerates energy production but also generates biosynthetic precursors [53, 54]. The resulting lactate efflux acidifies the TME, creating a hostile milieu that suppresses cytotoxic T cell function while promoting M2 polarization of TAMs [55, 56]. Recent investigations into the UBR5/RORA/SPLUNC1 axis reveal its critical role in amplifying this immunosuppressive cascade, particularly in NPC, where UBR5 silencing attenuates GPR132-mediated M2 macrophage reprogramming and glycolytic flux in preclinical models [57]. This axis exemplifies how tumor-intrinsic metabolic alterations propagate microenvironmental remodeling, establishing feedforward loops that sustain proliferation under nutrient stress.

Beyond glucose metabolism, tumor cells exploit amino acid and lipid networks to fortify survival mechanisms. Glutamine serves dual roles as a nitrogen donor for nucleotide synthesis and a carbon source for TCA cycle anaplerosis, with its catabolism generating α-ketoglutarate to mitigate oxidative damage [58, 59]. Parallel lipid adaptations emerge under nutrient deprivation: fatty acid β-oxidation becomes a primary energy reservoir, while de novo lipogenesis—orchestrated by mSREBP-1—supports membrane biosynthesis and chemoresistance [60, 61]. In CRC, FUT2-mediated fucosylation stabilizes mSREBP-1 and enables YAP1 nuclear translocation, thereby coupling hexosamine pathway activity to lipogenic programming—a dependency underscored by FUT2 knockout studies showing suppressed tumorigenesis and metastasis [62]. These metabolic liaisons are further modulated by lactate, which induces epigenetic reprogramming in CAFs to secrete pro-invasive matrix components, effectively engineering a metastatic niche [63].

### Metabolic reprogramming influences tumor invasion and metastasis

Metabolic reprogramming drives malignant progression through dynamic adaptations that sustain tumor bioenergetics and microenvironmental crosstalk. In lung adenocarcinoma (LUAD), mitochondrial ribosomal protein MRPL12 emerges as a master regulator, orchestrating mitochondrial OXPHOS addiction to fuel organoid formation and metastatic dissemination [64]. This OXPHOS dominance generates ATP and citrate pools that stabilize HIF-2α, creating a feedforward loop that amplifies tumor invasiveness while suppressing immunogenic cell death [65]. Parallel to mitochondrial adaptations, TCTN1 scaffolds fatty acid oxidation (FAO) machinery in melanoma, where its interaction with HADHA/HADHB activates p38/MAPK signaling to drive EMT and cancer stemness—a vulnerability exploitable by fluprostenol-mediated complex disruption [66].

Glucose metabolism remains central to tumor plasticity, with the Warburg effect dominating normoxic niches to generate lactate-fueled acidic microdomains that paralyze T cell surveillance [67]. Hypoxic compartments paradoxically engage TCA cycle rewiring, where BNIP3-mediated mitophagy clears dysfunctional mitochondria while enhancing OXPHOS efficiency in uveal melanoma (UM), enabling metastatic escape through PDK4-mediated pyruvate shunting [68]. This metabolic bifurcation—glycolytic versus oxidative phenotypes—creates therapeutic dilemmas, as BNIP3 inhibition may suppress metastasis but exacerbate glycolytic dependency in primary tumors.

Beyond tumor-intrinsic mechanisms, CAFs architect a nutrient-rich TME through alanine secretion and lactate shuttle systems that sustain tumor anabolism under glutamine deprivation. CAF-derived exosomes transport miR-105-5p to suppress c-Myc ubiquitination in adjacent tumor cells, amplifying glycolytic flux and chemoresistance—a symbiosis disrupted by miR-105-5p sponges in preclinical models [69]. Such metabolic interdependencies highlight the imperative for dual targeting of tumor-stromal axes, exemplified by ongoing trials combining glutaminase inhibitors (telaglenastat) with PD-L1 blockade in OXPHOS-high malignancies [20, 70].

### The impact of metabolic reprogramming on drug resistance in tumors

The TME serves as a pivotal regulator of malignant progression, where stromal-immune-metabolic crosstalk engineers an ecosystem permissive for therapeutic resistance and metastatic dissemination. CAF exemplify this orchestration, secreting lactate that acidifies extracellular pH to paralyze cytotoxic T lymphocyte (CTL) granzyme B activity while inducing PD-1 expression on tumor-infiltrating CD8^+^ T cells [71, 72]. Metabolite-mediated immunosuppression creates self-reinforcing circuits. Lactate accumulation activates HIF-1α in dendritic cells (DCs), blunting antigen presentation through CD83 downregulation and IL-10 overproduction [73]. Concurrently, kynurenine accumulation from IDO1-positive CAFs expands regulatory T cells (Tregs) via aryl hydrocarbon receptor (AhR) signaling, establishing anergy niches around metastatic deposits [74]. These adaptations are further amplified by extracellular vesicle trafficking, where CAF-derived Annexin A6 primes pre-metastatic lung niches through LPAR1-mediated YAP activation in resident fibroblasts [75].

Therapeutic targeting of TME metabolism demands spatiotemporal precision. Lactate dehydrogenase A (LDHA) inhibitors demonstrate restored CTL infiltration in head and neck cancer models, particularly when combined with anti-CSF1R to deplete protumor macrophages [76]. Similarly, FASN inhibitors disrupt lipid raft formation on Treg membranes, sensitizing tumors to PD-1 blockade in mismatch repair-proficient CRCs [77]. Emerging tools like hyperpolarized 13C-pyruvate MRI now enable real-time mapping of metabolic vulnerabilities, guiding personalized combinations of OXPHOS inhibitors with angiogenesis modulators [78].

### Tumor-targeted therapy based on metabolic reprogramming

The advent of molecularly targeted therapies has reshaped therapeutic paradigms in oncology, with metabolic reprogramming emerging as both a vulnerability and resistance mechanism across malignancies. TME remodeling orchestrates metabolic plasticity through stromal-immune crosstalk, wherein CAFs secrete oncometabolites like lactate and kynurenine that subvert CD8^+^ T cell effector functions while licensing PD-L1 expression on tumor cells [79, 80]. This metabolic hijacking converges on master regulators—HIF-1α stabilization amplifies glycolytic flux via LDHA upregulation [81], while mTORC1 activation redirects glutamine carbon into nucleotide biosynthesis through CAD phosphorylation [82]. Such adaptations create therapeutic dependencies: VHL-mutant ccRCC exhibit exquisite sensitivity to HIF-2α antagonists [83], whereas IDH1-mutant gliomas succumb to AG-120-mediated 2-hydroxyglutarate depletion [84].

Natural products are redefining adjuvant therapeutic strategies by targeting metabolic nodes with polypharmacological precision. Flavonoids like quercetin inhibit GLUT1-mediated glucose uptake while suppressing SREBP-1-dependent lipogenesis, synergizing with anti-PD-1 to reverse T cell exhaustion in melanoma models [85]. Similarly, terpenoid derivatives disrupt mitochondrial electron transport chain complexes, forcing OXPHOS-addicted tumors into metabolic crisis without compromising normal cell viability [86]. These agents counterbalance the limitations of single-pathway inhibitors—their pleiotropic mechanisms circumvent compensatory pathway activation, a key resistance driver in ACLY inhibitor trials.

## Metabolic reprogramming in RCC

RCC is increasingly recognized as a quintessential metabolic disease driven by multilayered reprogramming of glucose, lipid, and mitochondrial bioenergetics [87, 88]. Multi-omics profiling reveals that over 70% of ccRCC-associated mutations directly impact metabolic enzymes or regulators, creating a unique dependency on aerobic glycolysis (Warburg effect), aberrant fatty acid synthesis, and truncated TCA cycles [88]. This metabolic plasticity not only fuels tumor proliferation but also establishes grade-specific vulnerabilities, as evidenced by differential responses to glycolysis inhibitors in low- vs. high-grade tumors [89].

### Glucose metabolism

**Fig. 1:**
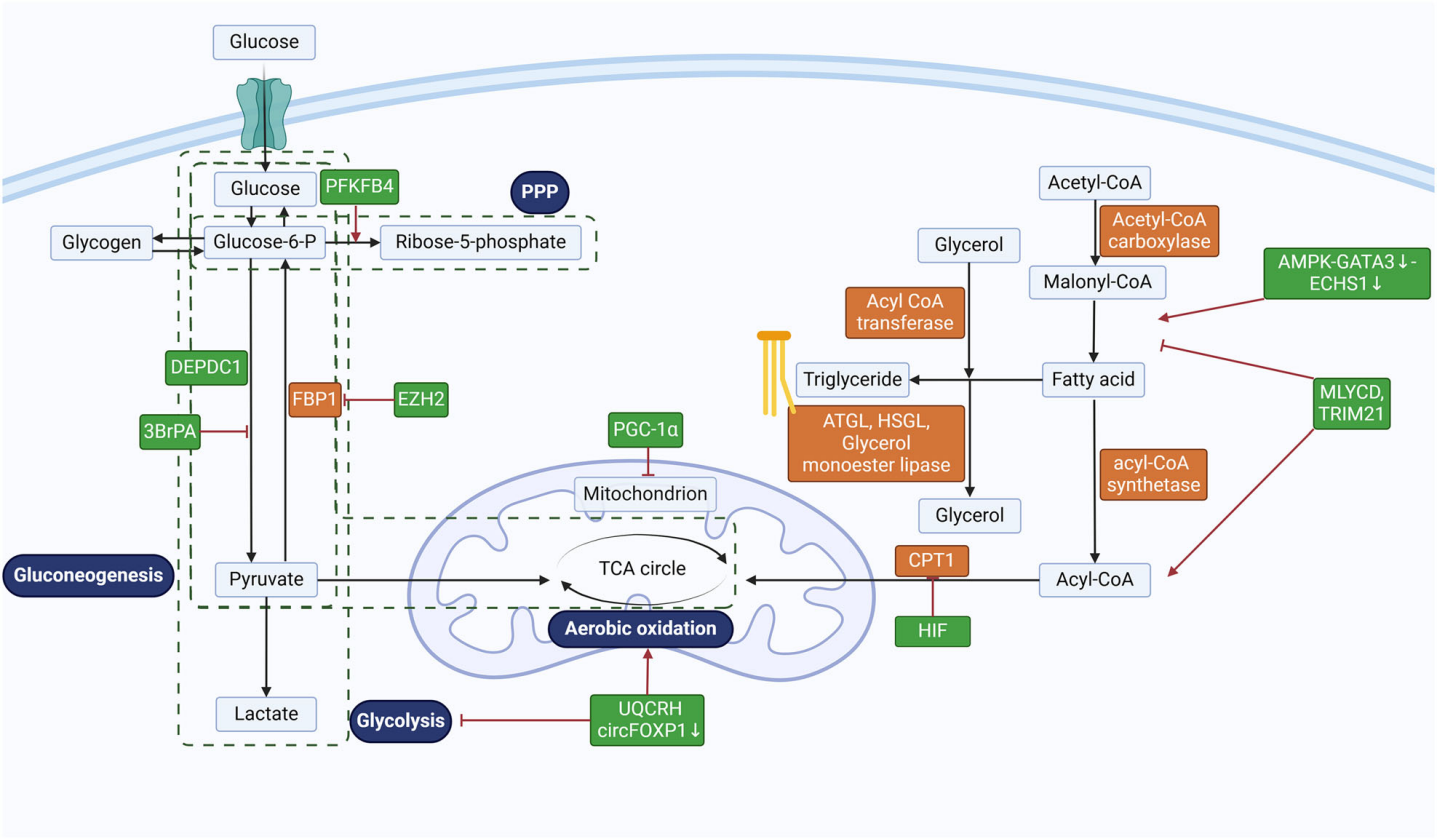
Glucose metabolism and lipid metabolism reprogramming in RCC. The balance between gluconeogenesis and glycolysis is intricately regulated during kidney cancer progression. The reciprocal regulatory interaction between FBP1 and EZH2, key components of gluconeogenic pathways, has been identified as a potential therapeutic target for renal cell carcinoma (RCC). In contrast, the glycolysis-associated gene DEPDC1 plays a pivotal role in driving malignant progression and drug resistance in RCC, suggesting that glycolysis inhibitors such as 3BrPA may offer promising targeted therapeutic options for ccRCC. Additionally, PFKFB4 has been shown to mediate resistance to sunitinib by enhancing the pentose phosphate pathway (PPP). Meanwhile, ubiquinone-cytochrome c reductase hinge protein (UQCRH) and circFOXP1 influence RCC progression through their impact on the Warburg effect. RCC progression is significantly influenced by key enzymes involved in the fatty acid metabolic pathway. Malonyl coenzyme A decarboxylase (MLYCD) and E3 ubiquitin ligase TRIM21 suppress tumor progression by inhibiting fatty acid synthesis and promoting fatty acid oxidation. Conversely, the AMPK-GATA3-ECHS1 pathway facilitates fatty acid synthesis and supports cancer cell proliferation. Additionally, hypoxia-inducible factors (HIFs) inhibit fatty acid oxidation by downregulating carnitine palmitoyltransferase 1 (CPT1). Created by Biorender.com. RCC renal cell carcinoma, UQCRH ubiquinone-cytochrome c reductase hinge protein, PPP pentose phosphate pathway, MLYCD malonyl coenzyme A decarboxylase, HIFs hypoxia-inducible factors, CPT1 carnitine palmitoyltransferase 1.

Glucose metabolism in ccRCC is driven by a complex interplay of HIFs, epigenetic modifications, and metabolic plasticity that collectively sustain tumor growth under diverse microenvironmental conditions (Fig. 1). The roles and mechanism of glucose metabolism in RCC were showed in Table 1. The canonical VHL-HIF-2α axis lies at the core of this reprogramming, where HIF-2α stabilization transcriptionally activates pyruvate dehydrogenase kinase (PDK), phosphorylating and inactivating pyruvate dehydrogenase (PDH) to redirect pyruvate into lactate production rather than mitochondrial oxidation [90]. This metabolic shift is corroborated by elevated lactate/pyruvate ratios in ccRCC patient urine, which normalize following tumor resection [91]. Concurrently, epigenetic silencing of the gluconeogenic enzyme fructose-1,6-bisphosphatase (FBP1) by polycomb repressive complex 2 (PRC2) reinforces glycolytic dependency [92]. EZH2-mediated promoter hypermethylation represses FBP1, while FBP1 itself inhibits PRC2 activity through direct interaction with EZH2—a tumor-suppressive feedback loop disrupted by genotoxic agents that stabilize EZH2. Restoring FBP1 expression reverses these effects, suppressing tumorigenesis and nominating FBP1 as a therapeutic target.

**Table 1:** The roles and mechanism of glucose metabolism in RCC.

Target	Molecular mechanism	Biological function	Reference
HIF-2α/PDK-PDH axis	HIF-2α stabilization activates PDK, phosphorylates and inactivates PDH, diverting pyruvate to lactate production.	Redirects glucose metabolism from mitochondrial oxidation to aerobic glycolysis (Warburg effect).	[90]
FBP1	Epigenetic silencing by PRC2 (EZH2-mediated promoter hypermethylation) disrupts FBP1-PRC2 feedback loop.	Reinforces glycolytic dependency; loss of FBP1 promotes tumorigenesis.	[92]
G6PDH	Upregulation in low-grade ccRCC channels glucose-6-phosphate into the pentose phosphate pathway (PPP).	Supports NADPH production (antioxidant defense) and ribose-5-phosphate synthesis (nucleotide biosynthesis).	[89, 90]
UQCRH	Downregulated via promoter hypermethylation, exacerbating mitochondrial dysfunction.	Sustains Warburg metabolism; rescuing UQCRH restores oxidative phosphorylation.	[93]
circFOXP1/miR-423-5p	circFOXP1 sponges miR-423-5p to upregulate U2AF2.	Amplifies glycolytic flux, promoting tumor proliferation and invasion.	[94]
DEPDC1	Activates HIF1α via AKT/mTOR signaling under hypoxia.	Sustains glycolysis and confers resistance to tyrosine kinase inhibitors (TKIs).	[95]
3-Bromopyruvate (3BrPA)	Selectively depletes ATP in ccRCC cells with mitochondrial defects.	Induces apoptosis in glycolytic-dependent tumors; ineffective in oxidative or FH-mutant tumors.	[96]
Lactate/Pyruvate ratio	Elevated in ccRCC patient urine, normalizes post-tumor resection.	Biomarker of glycolytic activity and tumor burden.	[91]

The Warburg effect in ccRCC exhibits striking grade-dependent heterogeneity. High-grade tumors predominantly rely on aerobic glycolysis, whereas low-grade ccRCC paradoxically channels glucose-6-phosphate into the pentose phosphate pathway (PPP) via glucose-6-phosphate dehydrogenase (G6PDH) upregulation [90]. This PPP activation not only sustains NADPH pools to counteract oxidative stress but also provides ribose-5-phosphate for nucleotide synthesis, correlating with aggressive clinical behavior and poor progression-free survival. Such metabolic divergence underpins differential therapeutic vulnerabilities: 2-deoxy-D-glucose (2DG) preferentially disrupts lactate metabolism in high-grade tumors but impairs proliferation in low-grade ccRCC [89]. However, subtype-specific resistance complicates this approach, as evidenced by a case of FH-mutant papillary RCC-2 (HLRCC syndrome) that progressed despite mTORC1 inhibitor and 2DG therapy, underscoring the need for molecular stratification.

Molecular regulators of glycolytic flux further refine therapeutic opportunities. Ubiquinol-cytochrome c reductase hinge protein (UQCRH), frequently downregulated via promoter hypermethylation in ccRCC, exacerbates mitochondrial dysfunction and Warburg metabolism [93]. Restoring UQCRH in glycolytic-defective models rescues oxygen consumption and induces apoptosis, while its knockdown in already glycolytic cells shows minimal effects, highlighting context-dependent roles. Circular RNA FOXP1 (circFOXP1) amplifies glycolytic flux by sponging miR-423-5p to upregulate U2AF2, promoting proliferation and invasion—a pathway silenced by circFOXP1 knockdown [94]. At the single-cell level, DEPDC1 emerges as a master regulator of hypoxia adaptation, activating HIF1α via AKT/mTOR signaling to sustain glycolysis and confer tyrosine kinase inhibitor (TKI) resistance [95]. DEPDC1 silencing restores TKI sensitivity, with high DEPDC1 expression correlating with metastatic progression and poor prognosis.

Targeting these vulnerabilities requires precision. While 3-bromopyruvate (3BrPA) selectively depletes ATP and induces apoptosis in ccRCC cells with intact mitochondrial defects, its efficacy is diminished in tumors with intact oxidative metabolism or FH mutations [96]. This dichotomy emphasizes the importance of combinatorial approaches, such as pairing glycolysis inhibitors with HIF2α antagonists to simultaneously disrupt energy production and hypoxia adaptation. Collectively, these insights position glucose metabolism not as a static hallmark but as a dynamic, targetable axis in ccRCC, where molecular stratification will unlock the full potential of metabolic therapy.

### Mitochondria metabolism

Mitochondrial biogenesis and dysfunction are central to the metabolic reprogramming of ccRCC, where impaired energy production and apoptotic resistance converge to drive tumor progression. The roles and mechanism of mitochondria metabolism in RCC were dispalyed in Table 2. In this malignancy, hypoxia-inducible carbonic anhydrase IX (CA9) is markedly overexpressed, suppressing oxidative phosphorylation while enhancing glycolytic flux—a hallmark of the Warburg effect [97]. Silencing CA9 not only upregulates mitochondrial biogenesis-related proteins but also induces spermidine accumulation, which inhibits proliferation and disrupts extracellular matrix interactions, thereby impairing tumor cell motility. Beyond CA9, mitochondrial complex I deficiency emerges as another critical defect, driven by NDUFA4L2 overexpression [98]. This loss forces cancer cells to rely on reductive carboxylation of glutamine-derived α-ketoglutarate for lipogenesis, bypassing canonical TCA cycle activity. Restoring complex I function through NDUFA4L2 knockdown reverses this metabolic adaptation, increasing ROS production and triggering hypoxia-selective apoptosis.

**Table 2:** The roles and mechanism of mitochondria metabolism in RCC.

Target	Molecular mechanism	Biological function	Reference
CA9	Overexpressed under hypoxia; suppresses oxidative phosphorylation (OXPHOS) and enhances Warburg glycolysis.	Promotes glycolytic dominance, tumor cell motility, and extracellular matrix interactions. Silencing CA9 induces mitochondrial biogenesis and spermidine accumulation, inhibiting proliferation.	[97]
NDUFA4L2	Overexpression drives mitochondrial complex I deficiency, forcing reliance on reductive carboxylation of glutamine-derived α-ketoglutarate.	Bypasses TCA cycle activity for lipogenesis; knockdown restores OXPHOS, increases ROS, and triggers hypoxia-selective apoptosis.	[98]
Dichloroacetate (DCA)	Inhibits PDK, reactivating OXPHOS in VHL-deficient ccRCC by suppressing HIF-mediated glycolysis.	Restores mitochondrial membrane potential, reduces angiogenesis, and inhibits HIF transcriptional activity.	[99]
FH deficiency	Loss of FH leads to fumarate accumulation, inhibiting PHDs and stabilizing HIF-α under normoxia (“pseudohypoxia”).	Sustains VEGF-driven angiogenesis and creates a self-reinforcing loop between metabolic dysregulation and vascular remodeling.	[100]
HIF1α-GPD1 feedback loop	HIF1α transcriptionally downregulates GPD1, suppressing mitochondrial respiration. GPD1 overexpression inhibits PHD3, stabilizing HIF1α and activating AMPK phosphorylation.	Sustains lipid metabolic rewiring and tumor growth; reactivating GPD1 may restore lipid homeostasis.	[101]

Therapeutic strategies to reverse mitochondrial dysfunction show promise. Dichloroacetate (DCA), a PDK inhibitor, reactivates oxidative phosphorylation in VHL-deficient ccRCC by suppressing HIF-mediated glycolytic dominance [99]. Preclinical models demonstrate DCA’s dual effects: restoring mitochondrial membrane potential while reducing tumor angiogenesis and HIF transcriptional activity. These findings align with observations in fumarate hydratase (FH)-deficient ccRCC subtypes, where FH loss leads to fumarate accumulation—a potent inhibitor of prolyl hydroxylases (PHDs) that stabilizes HIF-α even under normoxia [100]. This “pseudohypoxic” state perpetuates VEGF-driven angiogenesis, creating a self-reinforcing loop between metabolic dysregulation and vascular remodeling.

Metabolic perturbations in ccRCC are further amplified by the HIF1α-GPD1 feedback loop. GPD1 downregulation, transcriptionally enforced by HIF1α, suppresses mitochondrial respiration and promotes lipid metabolic rewiring. Conversely, GPD1 overexpression inhibits PHD3, stabilizing HIF1α and activating AMPK phosphorylation—a vicious cycle that sustains tumor growth [101]. Clinically, the interplay between these pathways underscores the potential of targeting mitochondrial-metabolic crosstalk. For instance, combining DCA with HIF2α inhibitors may synergistically disrupt both glycolytic and redox adaptations, while therapies reactivating GPD1 could restore lipid homeostasis. Collectively, these advances position mitochondrial dysfunction not merely as a bystander effect but as a linchpin of ccRCC pathogenesis, offering multifaceted opportunities for therapeutic intervention.

### Lipid metabolism

Fatty acid oxidation (FAO) and lipid synthesis are pivotal metabolic processes in ccRCC, providing energy, membrane components, and signaling molecules to fuel tumor progression (Fig. 1). The roles and mechanism of lipid metabolism in RCC were listed in Table 3. Recent studies reveal that ccRCC exhibits an adipocyte-like phenotype characterized by neutral lipid droplet (LD) accumulation, driven by Annexin A3 (AnxA3) isoform switching (reduced 36-kDa/33-kDa ratio), which promotes LD biogenesis via dysregulated vesicular trafficking [100]. This grade-dependent lipid dependency is further evidenced by the reliance of high-grade tumors on FAO—demonstrated by etomoxir sensitivity—whereas low-grade ccRCC depends on de novo lipogenesis [89]. Such metabolic plasticity underscores the need for stratified therapeutic strategies.

**Table 3:** The roles and mechanism of lipid metabolism in RCC.

Target	Molecular mechanism	Biological function	Reference
Annexin A3 (AnxA3)	Isoform switching (reduced 36-kDa/33-kDa ratio) dysregulates vesicular trafficking, promoting lipid droplet (LD) biogenesis.	Drives adipocyte-like phenotype with LD accumulation; grade-dependent lipid dependency (high-grade: FAO; low-grade: de novo lipogenesis).	[89, 100]
MUC1	Overexpression upregulates FASN and LDL receptors, amplifying lipid synthesis and storage.	Coordinates Warburg metabolism and cisplatin resistance; soluble MUC1 (CA15-3) correlates with poor progression-free survival.	[102, 103]
TRIM21	Degrades SREBF1 to suppress lipogenic enzymes (e.g., FASN, ACC).	Inhibits de novo lipogenesis; synergizes with FAO suppression for metabolic targeting.	[104]
Chemerin	Inhibits FAO via GPR1/CMKLR1 receptors, sustaining HIF-2α activation.	Maintains pseudohypoxic signaling; promotes lipid storage and tumor survival.	[104]
HIF-2α/PLIN2	HIF-2α-driven PLIN2 overexpression stabilizes LDs and protects against ER stress.	Enhances lipid storage and tumor resilience; HIF-2α suppression induces ER stress-mediated death.	[105]
CPT1A	Downregulation impairs mitochondrial FAO, exacerbating lipid accumulation.	Creates vulnerability to FAO reactivation therapies (e.g., etomoxir).	[106]
AMPK-GATA3-ECHS1 axis	ECHS1 deficiency triggers fatty acid and BCAA accumulation, activating AMPK-mTORC1 signaling.	Drives proliferation via metabolic crosstalk; links lipid metabolism to growth signaling.	[107]
MLYCD	Restoration depletes malonyl-CoA, disrupting lipid homeostasis.	Induces ferroptosis; synergizes with mitochondrial-targeted therapies.	[108]

A subset of aggressive ccRCC is defined by MUC1 overexpression, which coordinates the upregulation of FASN and LDL receptors, amplifying lipid synthesis and storage [102, 103]. MUC1 knockdown reverses Warburg metabolism, depletes lipid stores, and sensitizes cells to cisplatin, positioning it as a master regulator of metabolic reprogramming. Clinically, soluble MUC1 (CA15-3) serves as an independent biomarker for progression-free survival, highlighting its translational relevance [102]. These findings align with the role of other lipid regulators, such as TRIM21, which degrades sterol regulatory element-binding protein 1 (SREBF1) to suppress lipogenic enzymes, and chemerin, which inhibits FAO via GPR1/CMKLR1 receptors to sustain HIF-2α activation [104].

Lipid metabolism in ccRCC is further modulated by HIF-2α-driven pathways. Perilipin 2 (PLIN2), overexpressed in pseudohypoxic tumors, stabilizes LDs and protects against endoplasmic reticulum (ER) stress, while HIF-2α suppression reduces PLIN2 expression and enhances ER stress-induced cell death [105]. Concurrently, carnitine palmitoyltransferase 1 A (CPT1A) downregulation impairs mitochondrial FAO, exacerbating lipid accumulation—a vulnerability exploitable by FAO reactivation therapies [106]. The AMPK-GATA3-ECHS1 axis exemplifies metabolic crosstalk: enoyl-CoA hydratase 1 (ECHS1) deficiency triggers fatty acid and branched-chain amino acid accumulation, activating AMPK-mTORC1 signaling to drive proliferation [107].

Emerging therapeutic strategies target these interconnected pathways. Dichloroacetate, a PDK inhibitor, restores mitochondrial function and suppresses HIF activity, while MLYCD restoration disrupts lipid homeostasis via malonyl-CoA depletion, inducing ferroptosis [108].

### Amino acid metabolism

**Fig. 2:**
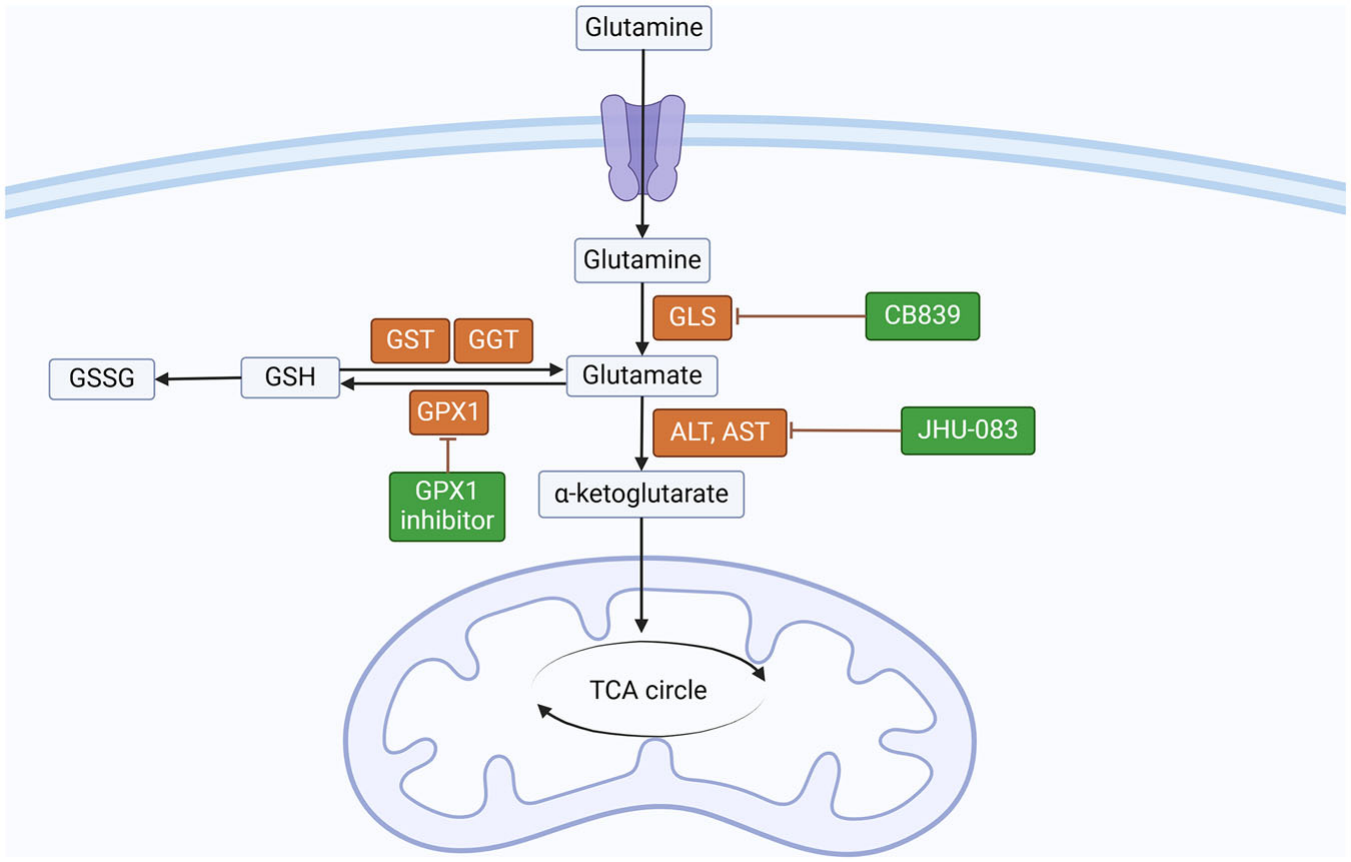
Amino acid metabolism reprogramming in RCC. Glutamine metabolism plays a pivotal role in the metabolic reprogramming of renal cell carcinoma (RCC). The aminotransferase inhibitor JHU-083 has been shown to significantly suppress tumor growth, while the glutaminase (GLS) inhibitor CB-839 similarly demonstrated a marked ability to inhibit the growth of xenograft tumors. Created by Biorender.com. RCC renal cell carcinoma, GLS glutaminase, GSH glutathione, GSSG glutathione disulfide, ALT Alanine aminotransferase, AST Aspartate aminotransferase, GST glutathione S-transferase, GPX Glutathione peroxide.

Glutamine metabolism plays a pivotal role in the metabolic reprogramming of tumors, providing key intermediates for energy production, biosynthesis, and redox balance. In cancer cells, glutamine is often consumed at higher rates to fuel anabolic processes, particularly under conditions of increased cell proliferation and metabolic stress. This makes glutamine metabolism an attractive therapeutic target in RCC, where alterations in metabolic pathways contribute to tumor growth and progression. Recent studies underscore the critical role of glutamine metabolism in RCC, revealing its potential as a therapeutic target (Fig. 2, Table 4). RCCs with VHL gene mutations exhibit unique metabolic characteristics, especially in the oxidative and reductive metabolism of glutamate. This metabolic alteration allows VHL-deficient RCC cells to utilize glutamine for producing essential metabolic intermediates, facilitating their rapid growth. Inhibition of isocitrate dehydrogenase-1 (IDH1) or -2 (IDH2) disrupts the TCA cycle, thereby curtailing tumor growth by limiting biosynthesis and energy production intermediates. While glutaminase inhibitors have shown limited success in tumor inhibition, the aminotransferase inhibitor JHU-083 has been notably effective in reducing tumor growth in mouse models, highlighting the vital role of aminotransferases in RCC progression [109]. Further research indicates that the long non-coding RNA MIR4435-2HG, highly expressed in fumarate hydratase (FH)-deficient RCC, is pivotal in glutamine metabolism. MIR4435-2HG interacts with STAT1 to transcriptionally activate the key glutaminase enzyme GLS1, positioning GLS1 as a potential therapeutic target for FH-deficient RCC [110]. Inhibitors such as CB-839, which target GLS1, have significantly suppressed xenograft tumor growth, suggesting that GLS1 inhibition could be an effective strategy for this RCC subtype.

**Table 4:** The roles and mechanism of amino acid metabolism in RCC.

Target	Molecular mechanism	Biological function	Reference
GLS1	Long non-coding RNA MIR4435-2HG interacts with STAT1 to transcriptionally activate GLS1, promoting glutamine hydrolysis.	Drives glutamine dependency in FH-deficient RCC; GLS1 inhibition (e.g., CB-839) suppresses tumor growth.	[110]
JHU-083	Aminotransferase inhibitor blocking nitrogen flux from glutamine to biosynthetic pathways.	Reduces tumor growth in preclinical models; highlights aminotransferase dependency.	[109]
IL-23-Treg axis	Glutamate depletion by tumor cells triggers TAM-derived IL-23 secretion, promoting Treg proliferation and suppressing cytotoxic T cells.	Facilitates immune evasion; IL-23 pathway inhibition enhances anti-PD-1 efficacy.	[111]
HIF-mediated reductive carboxylation (RC)	HIF activation in VHL-deficient RCC induces RC to generate citrate from glutamine-derived α-KG under hypoxia.	Supports tumor survival in hypoxic niches; citrate restoration inhibits RC.	[112]
MYC	MYC overexpression amplifies glutaminolysis, increasing glutamate availability for biosynthesis.	Drives proliferation in MYC-driven RCC; glutaminase inhibition slows tumor progression.	[113]
ASS1	Epigenetic activation of ASS1 in metastatic ccRCC enhances arginine synthesis via the urea cycle.	Supports arginine-dependent tumor growth and metastasis.	[114]
xCT transporter/GGT1	ChRCC relies on cystine uptake via xCT to maintain glutathione; GGT1 deficiency impairs glutathione synthesis.	Induces ferroptosis vulnerability; GGT1 overexpression suppresses ChRCC growth.	[115]
PLCG2/IP3/Ca^2+^/PKC pathway	Regulates endocytic uptake of amino acids from extracellular macromolecules in nutrient-poor environments.	Sustains ChRCC survival; pathway inhibition disrupts amino acid uptake and tumor adaptation.	[116]

In ccRCC, heightened glutamate metabolism is linked to poor prognosis. Tumor cells consume glutamate, leading to its local depletion, prompting TAMs to secrete IL-23. IL-23 fosters Treg proliferation and suppresses cytotoxic T cell function, aiding immune evasion and tumor progression. Inhibiting the IL-23 pathway has extended survival in mouse models and enhanced anti-PD-1 immunotherapy efficacy, making the glutamate metabolism-IL-23-Treg axis a promising immunotherapeutic target [111]. Moreover, HIFs are crucial in RCC, particularly in VHL-deficient tumors, where they induce reductive carboxylation (RC), a metabolic adaptation supporting tumor survival under hypoxia. Restoring intracellular citrate levels can inhibit RC, suggesting that targeting both HIFs and glutamate metabolism might be therapeutically beneficial in VHL-deficient RCC [112]. Additionally, MYC overexpression in RCC amplifies glutaminolysis, increasing glutamate availability for biosynthesis and energy production. Inhibition of glutaminase in MYC-driven RCC models has slowed tumor progression, indicating that targeting glutamine metabolism could be a viable therapeutic strategy for MYC-driven cancers [113].

Targeting key enzymes in glutamine metabolism, such as GLS1, or modulating immune responses through the glutamate-IL-23-Treg axis, may lead to novel and personalized RCC treatments, ultimately improving patient outcomes [111]. In ccRCC, metabolic reprogramming prominently features the alteration of branched-chain amino acid (BCAA) metabolism. Typically, BCAAs such as leucine, isoleucine, and valine are metabolized in the mitochondria to produce crucial metabolic intermediates. However, in ccRCC, the suppression of BCAA metabolism enhances the flexibility of the cancer cells’ metabolic network. These cancer cells exploit nitrogen from BCAAs to synthesize essential amino acids like aspartate and arginine, which are vital for tumor growth and survival. The epigenetic activation of the urea cycle enzyme argininosuccinate synthase (ASS1) in metastatic ccRCC tumors boosts arginine production, an amino acid critical for cell growth and metastasis [114]. These findings suggest that targeting amino acid metabolism, particularly the urea cycle, could be a promising therapeutic strategy in ccRCC. Conversely, ChRCC presents a distinct metabolic profile with a specific susceptibility to ferroptosis, a regulated form of cell death triggered by lipid peroxidation. ChRCC cells heavily depend on cystine uptake via the xCT transporter to maintain cellular glutathione levels, which are essential for oxidative stress defense. The expression of gamma-glutamyltransferase 1 (GGT1), a key enzyme for glutathione synthesis, is significantly reduced in ChRCC, compromising their ability to maintain redox balance and limiting proliferation. Remarkably, overexpressing GGT1 can restore glutathione synthesis, reduce cystine uptake, and suppress ChRCC cell growth. This suggests that targeting the xCT transporter or inducing ferroptosis could be viable therapeutic approaches for ChRCC [115]. Further studies on ChRCC have revealed additional metabolic adaptations, including a reliance on aerobic glycolysis (the Warburg effect) for energy production and the suppression of gluconeogenesis. ChRCC cells also depend on the endocytic uptake of amino acids from extracellular macromolecules, aiding their survival in nutrient-poor environments. The PLCG2/IP3/Ca^2+^/PKC signaling pathway is crucial in this adaptation, and inhibiting this pathway disrupts amino acid uptake, impairing the tumor cells’ metabolic adaptation and survival. These insights identify the PLCG2/IP3/Ca^2+^/PKC axis as a promising therapeutic target in ChRCC, highlighting new metabolic mechanisms of this RCC subtype [116].

### Metabolic-immune crosstalk in the TME of RCC

RCC, particularly ccRCC, is characterized by a profoundly immunosuppressive TME that dynamically interacts with metabolic reprogramming to drive therapeutic resistance (Table 5). As one of the most immune-infiltrated solid tumors, ccRCC exhibits dense infiltration of CD8^+^ T cells, macrophages, and mast cells, yet these populations are functionally compromised through multiple mechanisms [117, 118]. CD8^+^ T cells in ccRCC patients display defective anti-apoptotic and proliferative capacity due to miR-29b/miR-198-mediated suppression of JAK3 and MCL-1—a dysfunction reversible via miRNA inhibition [119]. Concurrently, the kynurenine (KYN) pathway is hyperactivated in ccRCC, with elevated KYN-to-tryptophan ratios (KTR) correlating with aggressive phenotypes and poor survival (5-year CSS: 76.9% vs. 92.3% for high vs. low KTR, *P* < 0.0001). This immunosuppressive axis not only depletes intratumoral tryptophan but also generates KYN metabolites that directly inhibit T cell function and promote regulatory T cell expansion [120].

**Table 5:** Metabolic-immune crosstalk in the TME of RCC.

Target	Molecular mechanism	Biological function	Reference
miR-29b/miR-198	Suppress JAK3 and MCL-1 expression in CD8^+^ T cells.	Impairs anti-apoptotic and proliferative capacity of CD8^+^ T cells; reversible via miRNA inhibition.	[119]
Kynurenine (KYN) pathway	Elevated KYN-to-tryptophan (KTR) ratio depletes tryptophan and generates immunosuppressive metabolites.	Inhibits T cell function, promotes Treg expansion; correlates with poor survival (5-year CSS: 76.9% vs. 92.3%, *P* < 0.0001).	[120]
Pentraxin-3 (PTX3)	Activates classical complement pathway (C1q/C3aR/C5aR) and upregulates CD59 to evade complement lysis.	Fosters pro-angiogenic niche (VEGF/IL-8 enrichment); drives M2 macrophage polarization and CD8^+^ T cell exclusion.	[121, 122]
Bevacizumab	Inhibits VEGF and reduces microvascular density; depletes CD68^+^ macrophages and tryptase+ mast cells.	Disrupts stromal pro-angiogenic factor release; synergizes with immune modulation.	[118]
Serum metabolomics	Baseline metabolic profiles predict immune checkpoint inhibitor (ICI) efficacy (>80% accuracy).	Enables patient stratification for personalized immunotherapy.	[123]
C3aR/C5aR complement axis	Inhibition blocks angiogenesis and restores CD8^+^ T cell infiltration.	Dual therapeutic node: targets angiogenesis and immunosuppression.	[124]
TME nutrient competition	Extracellular acidosis and nutrient scarcity drive CD8^+^ T cell exhaustion.	Promotes T cell dysfunction; tumor-associated fibroblasts secrete complement factors to sustain immunosuppression.	[125]

Metabolic crosstalk further shapes the angiogenic and inflammatory landscape. Pentraxin-3 (PTX3), overexpressed in ccRCC but absent in normal kidney, activates the classical complement pathway (C1q/C3aR/C5aR) while upregulating CD59 to evade complement-mediated lysis [121]. This dual role fosters a pro-angiogenic niche enriched with VEGF and IL-8, while MUC1-overexpressing tumors amplify PTX3-driven complement activation, correlating with M2 macrophage polarization, mast cell infiltration, and CD8^+^ T cell exclusion [122]. Such metabolic-immune synergy is exemplified by bevacizumab’s mechanism: beyond directly inhibiting VEGF, it reduces microvascular density and depletes CD68^+^ macrophages/tryptase^+^ mast cells in the TME, disrupting stromal pro-angiogenic factor release [118].

Therapeutic strategies must address this intertwined biology. While immune checkpoint inhibitors (ICIs) have revolutionized advanced ccRCC management, response heterogeneity persists. Serum metabolomics reveals that baseline metabolic fingerprints predict ICI efficacy with >80% accuracy in solid tumors, suggesting potential for ccRCC patient stratification [123]. Combinatorial approaches—such as HIF-2α inhibitors with anti-PD-1 agents—may simultaneously target hypoxic signaling and immune checkpoints. Notably, the complement system emerges as a dual therapeutic node: inhibiting C3aR/C5aR could block angiogenesis while restoring CD8^+^ T cell infiltration, whereas CD59 blockade may enhance complement-dependent cytotoxicity [124].

Critical challenges remain in decoding spatial heterogeneity. Single-cell analyses reveal that nutrient competition and extracellular acidosis in the TME drive CD8^+^ T cell exhaustion, while tumor-associated fibroblasts secrete complement factors to sustain immunosuppression [125]. Integrating stromal-immune-metabolic biomarkers through multi-omics profiling could unlock personalized regimens—stratifying patients for angiogenesis inhibitors, ICIs, or emerging complement-targeted therapies.

### Other metabolism

Metabolic reprogramming in RCC involves significant alterations in metabolic pathways that support rapid tumor growth and survival within the tumor microenvironment. Various oncogenes and tumor suppressor genes play crucial roles in modulating cellular metabolism and influencing tumor progression (Table 6). Recent studies have identified TRIM65 as a novel oncogene highly expressed in RCC tissues and cell lines. TRIM65 promotes tumor cell proliferation by ubiquitinating and degrading the tumor suppressor gene BTG3, which alleviates G2/M cell cycle arrest, thereby advancing RCC through metabolic changes [126]. Additionally, elevated expression of FKBP51 in RCC is associated with a higher risk of metastasis; FKBP51 enhances tumor cell invasion and migration by promoting the autophagic degradation of TIMP3, contributing to the aggressive nature of RCC [127]. PAK4 has emerged as a promising therapeutic target in RCC. The development of the PROTAC-based drug PpD, which specifically targets PAK4, has effectively inhibited tumor cell proliferation and demonstrated strong synergistic effects when combined with PD-1 blockade immunotherapy, enhancing the immune response against RCC [128]. Furthermore, EIF3D expression is significantly elevated in sunitinib-resistant RCC cell lines. EIF3D interacts with GRP78 to prevent protein degradation, activating the unfolded protein response and inducing resistance to therapy, underscoring the importance of understanding molecular mechanisms of metabolic reprogramming in RCC to develop more effective treatments [129].

**Table 6:** The roles and mechanism of other metabolism in RCC.

Target	Molecular mechanism	Metabolic pathway	Biological function	Reference
TRIM65	Ubiquitinates and degrades tumor suppressor BTG3, alleviating G2/M cell cycle arrest.	Ubiquitin-proteasome system	Promotes RCC proliferation via metabolic reprogramming.	[126]
FKBP51	Promotes autophagic degradation of TIMP3 to enhance tumor invasion and migration.	Autophagy	Drives metastatic progression and aggressiveness in RCC.	[127]
PAK4	Targeted by PROTAC drug PpD, which inhibits PAK4 and synergizes with anti-PD-1 therapy.	Kinase signaling	Suppresses tumor proliferation and enhances immunotherapy response.	[128]
EIF3D	Interacts with GRP78 to stabilize proteins and activate unfolded protein response (UPR).	Protein homeostasis	Induces therapy resistance in sunitinib-resistant RCC.	[129]
OXPHOS (chRCC)	Reduced mtDNA content impairs ETC complex subunit expression and OXPHOS function.	Mitochondrial respiration	Characterizes chRCC, distinguishes it from benign renal oncocytoma; linked to GSH overproduction.	[130]
PGC-1α (ccRCC)	Suppressed via HIF/Dec1-dependent mechanism, reducing Tfam and mitochondrial function.	Mitochondrial biogenesis	Promotes Warburg effect; restoring PGC-1α rescues OXPHOS and enhances therapy sensitivity.	[131]
PFKFB4	Overexpressed in ccRCC; activates PPP via phosphorylation of NCOA3-FBP1 complex.	Pentose phosphate pathway (PPP)	Drives sunitinib resistance, tumor progression, and redox homeostasis.	[132]
ABL1 kinase (FH-deficient RCC)	Activated in FH-deficient tumors, enhancing glycolysis and NRF2-mediated antioxidant defenses.	Aerobic glycolysis and oxidative stress	Vandetanib inhibits ABL1, prolonging survival in aggressive FH-deficient RCC.	[133]

OXPHOS is a crucial metabolic process in eukaryotic cells, where electrons are transferred through the electron transport chain (ETC) in mitochondria to produce ATP, the cell’s primary energy currency. In the context of cancer, particularly RCC, dysregulation of OXPHOS is a significant aspect of metabolic reprogramming. This alteration contributes to tumor progression, modified energy metabolism, and resistance to therapy. Tumors typically shift their dependency from OXPHOS to glycolysis or other compensatory pathways to fulfill their increased energy requirements. Recent investigations have identified distinct molecular mechanisms driving OXPHOS dysregulation across various RCC subtypes, including ccRCC and chRCC [20].

In chRCC, the mechanisms of OXPHOS dysregulation differ significantly from those in benign renal oncocytoma (RO). Research indicates that chRCC is characterized by reduced protein levels of several ETC complex subunits, not caused by mutations in complex I (CI) genes. This reduction is mainly due to a decrease in mitochondrial DNA (mtDNA) content. The depletion of mtDNA negatively correlates with the protein and transcription levels of nuclear DNA-encoded ETC subunits, impairing OXPHOS function [130]. Additionally, chRCC cells exhibit elevated levels of glutathione (GSH), an antioxidant marker indicating oxidative stress. This increase in GSH is linked to mtDNA depletion, suggesting an alternative GSH production pathway distinct from RO. These insights highlight the unique biological traits of chRCC and offer potential diagnostic markers to distinguish malignant chRCC from benign RO, underscoring the importance of OXPHOS dysregulation in RCC subtypes.

In ccRCC, OXPHOS dysfunction is a hallmark of metabolic reprogramming, notably through the suppression of PGC-1α, a crucial regulator of mitochondrial biogenesis and energy metabolism. In VHL-deficient ccRCC cells, PGC-1α inhibition occurs via an HIF/Dec1-dependent mechanism, leading to decreased expression of Tfam (mitochondrial transcription factor A), which is essential for maintaining mitochondrial DNA and respiratory function. As a result, mitochondrial dysfunction is observed, promoting altered cellular metabolism and supporting the Warburg effect [131]. However, restoring PGC-1α expression in VHL-deficient ccRCC cells can rescue mitochondrial function, induce oxidative stress, and significantly suppress tumor growth. This reactivation of mitochondrial respiration also enhances the sensitivity of ccRCC cells to cytotoxic treatments, highlighting the therapeutic potential of modulating PGC-1α expression in ccRCC.

Recent studies have highlighted the critical role of specific metabolic enzymes in RCC, especially in ccRCC, the most common subtype. Among these enzymes, 6-phosphofructo-2-kinase/fructose-2,6-bisphosphatase 4 (PFKFB4) is particularly noteworthy. Research indicates that PFKFB4 is overexpressed in ccRCC, significantly contributing to resistance against the targeted therapy sunitinib [132]. PFKFB4 enhances the PPP, which is vital for nucleotide biosynthesis and maintaining cellular redox balance. Its overexpression correlates with higher tumor grade, advanced stage, and poor prognosis, suggesting its potential as a biomarker for disease progression. Despite the common deletion of the chromosome 3p region in ccRCC, where the PFKFB4 gene is located, PFKFB4 remains actively transcribed in ccRCC cells. This transcription is mainly regulated by HIF1α, a principal regulator of the cellular response to hypoxia. Silencing PFKFB4 in ccRCC cells significantly diminishes their proliferative capacity, migration, and wound healing abilities, underscoring its crucial role in tumor progression and metastasis. Further molecular investigations reveal that PFKFB4 phosphorylates nuclear receptor coactivator 3 (NCOA3), which then interacts with FBP1 to control the PPP, essential for sustaining the altered metabolic state in ccRCC and maintaining cellular redox homeostasis. Targeting PFKFB4 presents a promising therapeutic approach to counteract sunitinib resistance in RCC. Both in vitro and in vivo models demonstrate that inhibiting PFKFB4 can effectively reduce tumor growth and increase the sensitivity of ccRCC cells to treatment. These findings position PFKFB4 as a potential therapeutic target for overcoming resistance and enhancing treatment outcomes in RCC.

Oxidative stress refers to an imbalance between the production of ROS and the body’s ability to counteract these harmful entities, leading to cellular damage. In the context of cancer, oxidative stress serves a dual purpose: it can promote tumor growth through mechanisms such as DNA damage and inflammation, while also being a potential target for therapeutic interventions. Recent studies have highlighted the significance of oxidative stress in various cancers, particularly renal cancer, where it is closely linked to tumor progression and resistance to treatment. Renal cancer exhibits considerable heterogeneity, with its advancement influenced by various molecular pathways. Investigating oxidative stress and its molecular pathways in renal cancer not only enhances our understanding of tumor progression but also provides vital insights for developing new therapeutic strategies. Notably, individuals with germline mutations in fumarate hydratase (FH) face a heightened risk of aggressive renal cancer, for which effective treatment options are scarce [133]. Unbiased drug screening of cell lines from FH-deficient tumors has revealed that the tyrosine kinase inhibitor vandetanib demonstrates significant cytotoxicity both in vitro and in vivo. Mechanistic studies suggest that the efficacy of vandetanib is dependent on ABL1 kinase activity, which is activated in FH-deficient renal tumors, leading to enhanced aerobic glycolysis and NRF2-mediated antioxidant defenses. In xenograft models, an 8-week treatment with vandetanib substantially increased tumor-free survival to 13 months, indicating that ABL1 inhibition could be a viable clinical strategy for managing aggressive FH-deficient renal cancers and other glycolysis- and oxidative stress-driven tumors. Additionally, research has investigated the sensitivity of FH-deficient renal cancer cells to oxidative stress.

## Clinical application of metabolic reprogramming in RCC

Metabolic reprogramming is a central feature of RCC pathogenesis, with therapeutic strategies targeting dysregulated metabolic pathways emerging as a promising approach for combating this challenging malignancy. Several clinical trials have investigated drugs that modulate key metabolic pathways in RCC, and these therapies hold great potential for improving treatment outcomes. mTOR inhibitors such as everolimus and temsirolimus disrupt the anabolic machinery of RCC by blocking ribosomal biogenesis and inhibiting HIF-2α-driven angiogenic signaling. These agents have demonstrated median progression-free survival (PFS) improvements of 3–5 months compared to sunitinib in pivotal trials [134, 135]. However, compensatory activation of AGC kinases through mTORC2 signaling often limits the long-term efficacy of these treatments. This resistance mechanism is partially overcome by next-generation dual TOR kinase inhibitors (TORKinibs), which simultaneously inhibit DNA-PK to impair homologous recombination repair, potentially enhancing therapeutic durability.

Beyond targeting the mTOR axis, the glycolytic vulnerability of RCC is being explored with agents like 2-DG, which traps HK2 in abortive phosphorylation cycles. This leads to the depletion of ATP in VHL-deficient ccRCC cells, while sparing normal renal epithelial cells, thus exploiting the metabolic differences between cancerous and normal cells [136]. The glutamine addiction model is also gaining clinical traction, with the glutaminase inhibitor CB-839 showing promising phase Ib results when combined with cabozantinib. This combination targets NADPH regeneration and redox balance disruption in TSC1/2-mutant tumors, offering potential clinical benefit in this subset [137]. Furthermore, tumors with FH loss exhibit a heightened dependence on glutamine carbon flux for aspartate biosynthesis. Emerging biomarker strategies are now being developed to better stratify patients for metabolism-directed therapies. For instance, FDG-PET avidity (SUVmax >5) and circulating 2-hydroxyglutarate levels are being used as biomarkers to identify RCC patients who may benefit from specific metabolic interventions [138]. Early-phase clinical data show promising results, with objective response rates of up to 45% when combining the HIF-2α antagonist belzutifan with LDH-A inhibitors in glycolytic-high RCC subsets [139].

The future of metabolic reprogramming in RCC therapy lies in the continued exploration of combination strategies. Integrating metabolic modulators with immune checkpoint inhibitors, targeted therapies, and other emerging agents could enhance treatment efficacy and overcome resistance mechanisms. Additionally, a more refined understanding of the metabolic heterogeneity within RCC subtypes, combined with advanced biomarker strategies, will enable more personalized and effective treatments. Furthermore, the development of next-generation inhibitors targeting specific metabolic pathways like glycolysis, glutaminolysis, and mitochondrial metabolism will be crucial in advancing the clinical management of RCC.

## Challenges and future directions

The metabolic reprogramming observed in RCC presents several hurdles in both preclinical and clinical settings, yet it also opens up substantial opportunities for advancing therapeutic strategies. Despite significant advancements in understanding the molecular mechanisms driving RCC metabolism, key obstacles remain in translating these insights into effective therapeutic strategies. One of the most pressing challenges is the metabolic heterogeneity observed across RCC histological subtypes and within different intratumoral regions. This metabolic diversity not only reflects the complex interplay between tumor cells and the surrounding microenvironment but also contributes to therapeutic resistance. For example, ccRCC relies heavily on glycolysis and glutamine metabolism, whereas other RCC subtypes such as pRCC may exhibit distinct dependencies on fatty acid oxidation. This variability complicates the identification of universal metabolic vulnerabilities and necessitates a precision medicine approach in clinical trials. Future research and trials must focus on defining precise metabolic signatures for each RCC subtype to enable more targeted and individualized therapies.

The TME plays a critical role in shaping RCC metabolism and contributes to both therapeutic resistance and immune evasion [140–142]. The TME in RCC is highly complex, involving intricate interactions between tumor cells, CAFs, endothelial cells (ECs), and immune cells such as myeloid-derived suppressor cells (MDSCs). These stromal components significantly influence tumor metabolism, contributing to the establishment of immunosuppressive niches that hinder effective immune surveillance [143, 144]. Additionally, CAFs and ECs not only promote glycolytic flux but also drive lipid and amino acid metabolism, creating metabolic dependencies that tumors exploit for growth and survival [145, 146]. Therefore, TME-targeting strategies are essential for improving treatment outcomes, particularly in combination with immunotherapies. Clinical trials evaluating the use of glutaminase inhibitors, PFKFB3 inhibitors, or CAFs-targeting therapies could offer exciting avenues for overcoming TME-mediated resistance [147, 148]. However, the challenge remains in translating preclinical findings into the clinic, where the TME’s complexity may limit the efficacy of single-agent therapies.

Another critical challenge is the issue of adaptive resistance to single-agent therapies. Targeting specific metabolic pathways, such as glutaminase (GLS) or acetyl-CoA carboxylase (ACC), has shown promise in preclinical models; however, tumors often compensate by activating alternative metabolic pathways. For instance, inhibition of glycolysis can result in compensatory activation of the PPP or fatty acid oxidation, thereby maintaining tumor viability [149]. To address this, combination therapies targeting multiple metabolic networks simultaneously are necessary to prevent pathway compensation and overcome resistance. Furthermore, combining metabolic inhibitors with ICIs, such as PD-1 blockade, holds considerable potential to enhance anti-tumor immunity by reprogramming the immunosuppressive TME [150–152]. Early-phase clinical trials evaluating combinations of glutamine metabolism inhibitors and PD-1 blockade suggest potential synergistic effects in reinvigorating exhausted T cells and enhancing anti-tumor immunity [153, 154].

The emerging field of functional imaging and liquid biopsy also offers exciting possibilities for overcoming current challenges in RCC treatment. Advances in hyperpolarized 13C-pyruvate MRI and metabolomic profiling could allow real-time monitoring of tumor metabolism, facilitating the identification of early therapeutic responses and adjustments to treatment strategies [155]. Liquid biopsy platforms that detect oncometabolites, such as 2-HG, provide a non-invasive method for tracking treatment efficacy and assessing tumor metabolic status, further aiding in patient stratification and personalized therapy [156]. However, standardizing these techniques for widespread clinical use remains a challenge, and further validation of these biomarkers is necessary before they can be incorporated into routine clinical practice.

A crucial aspect of advancing clinical trials in RCC is the integration of multi-omics approaches, including genomics, transcriptomics, proteomics, metabolomics, and spatial biology. These techniques will allow for a comprehensive understanding of the molecular landscape of RCC and its metabolic rewiring. By combining single-cell metabolomics with spatial transcriptomics, researchers will be able to identify tumor-specific metabolic vulnerabilities and define the spatial heterogeneity of metabolic activity within RCC tumors. Such integrated approaches will be essential for identifying novel therapeutic targets and predicting clinical outcomes. However, the challenge lies in the complexity of interpreting these vast datasets and translating them into actionable insights for clinical trials.

As RCC’s metabolic pathways continue to be elucidated, the next step will be the design of clinical trials that incorporate these insights into therapeutic strategies. For example, trials could focus on combining metabolic inhibitors with immune checkpoint blockade to enhance the effectiveness of both therapies. Moreover, personalized treatment strategies based on metabolic profiling could be implemented, where patients are stratified based on their tumor’s unique metabolic vulnerabilities. The use of adaptive trial designs, where treatment regimens can be adjusted based on real-time biomarker data, will be crucial for optimizing therapeutic outcomes. Furthermore, targeting metabolic vulnerabilities in the stroma, such as those associated with CAFs or ECs, offers novel opportunities for therapeutic intervention. For example, lipid metabolism inhibitors could be evaluated in trials to target the pro-tumorigenic effects of CAFs, potentially enhancing the efficacy of chemotherapy or immunotherapy. Similarly, glutamine metabolism inhibitors may show promise in combination with ICIs in trials aimed at enhancing the anti-tumor immune response.

## Conclusion

Metabolic reprogramming is crucial in the progression of RCC, facilitating tumor growth and resistance to treatments. Key metabolic vulnerabilities such as the Warburg effect and altered lipid metabolism present potential intervention targets. Recent studies highlight the promise of targeting these pathways to slow RCC progression and improve outcomes, though challenges like tumor heterogeneity and metabolic flexibility hinder clinical application. Understanding the relationship between tumor metabolism and the immune system is vital for effectively combining metabolic inhibitors with therapies like immune checkpoint blockade. A personalized approach that considers patients’ unique metabolic profiles is essential. Advances in imaging, biomarkers, and selective metabolic inhibitors will enhance treatment strategies. Ultimately, while targeting metabolic pathways holds significant potential for RCC treatment, further research is necessary to address challenges and improve protocols, potentially transforming RCC management and patient survival rates.
